# Cortical thickness, surface area, and multisite pain: distinct patterns by sex in adolescence

**DOI:** 10.1186/s13293-026-00898-6

**Published:** 2026-04-06

**Authors:** Esmeralda Hidalgo-Lopez, Christel Portengen, Tristin Smith, Hannah C. Becker, Andrew Schrepf, Steven E. Harte, Adriene M. Beltz, Chelsea M. Kaplan

**Affiliations:** 1 Chronic Pain and Fatigue Research Center, Department of Anesthesiology, University of Michigan Medical School, 24 Frank Lloyd Wright Dr., Lobby M, Suite 3100, Ann Arbor, MI USA; 2 Department of Psychology, University of Michigan, 530 Church St, Ann Arbor, MI USA

**Keywords:** Multisite Pain, Brain Structure, Adolescence, Sex Differences, Pubertal Development

## Abstract

**Background:**

Multisite pain is common during adolescence and is influenced by sex-related neurobiological and developmental factors, but its developmental neural mechanisms are unclear.

**Methods:**

Utilizing data from the Adolescent Brain and Cognitive Development Study, we investigated the relation between cortical brain structure and multisite pain (assessed by youth self-reports of painful regions on a body map) in male (*N* = 3,299) and female (*N* = 2,844) adolescents aged 11–12 years. We focused on brain regions functionally linked to multisite pain (i.e., bilateral sensorimotor, cingulate, fronto-insular and inferior parietal cortex). We also explored the moderating role of pubertal status (assessed by the Pubertal Development Scale).

**Results:**

Findings revealed distinct brain structure-pain associations in male and female youth. Male youth exhibited an inverse linear relation between cortical thickness of the left pre- and postcentral gyri and number of pain sites. Female youth exhibited a non-linear relation between surface area of the right supramarginal gyrus and number of pain sites. Pubertal status moderated the cingulate cortical thickness-pain association in males; those in early puberty had an inverse relation between anterior and mid cingulate cortex thickness and pain sites, whereas this relation was positive in those beyond mid-puberty.

**Conclusion:**

This study provides valuable insights into the sex-dependent neural organization linked to adolescent pain.

**Supplementary Information:**

The online version contains supplementary material available at 10.1186/s13293-026-00898-6.

## Abstract (plain language summary)

Many adolescents, especially female adolescents, experience pain in multiple parts of their body, known as “multisite pain”. This type of pain is not related to injury, can be an early sign of lifelong pain, and is especially difficult to treat. In fact, this type of pain seems to stem directly from the brain. Yet, it is surprising that little is known about if, or how, the brains of adolescents with multisite pain differ from those without, or how sex-related biology (i.e., puberty) contributes to it. In this study, we looked at brain scans from 6,143 adolescents aged 11 to 12 years from the largest study of adolescent neurodevelopment in the world. We investigated how the structure of brain areas (that contribute to other pain conditions) relate to multisite pain. We found that male adolescents with multisite pain had alterations in brain regions involved in touch and movement (pre- and post-central gyri). Female adolescents with higher degrees of multisite pain (i.e., in three or more areas) had a larger surface area of a brain region related to body awareness and pain processing (supramarginal gyrus). Puberty also played a role, but most prominently for males: The relation between pain and the thickness of a brain area involved in attention and motivation (the cingulate) depended on how advanced they were in puberty. These findings help us understand how brain development and puberty contribute to sex differences in the experience of adolescent pain and will hopefully inform treatments that maximize wellbeing for all.

**Supplementary Information:**

The online version contains supplementary material available at 10.1186/s13293-026-00898-6.

## Highlights

- Adolescent multisite pain is influenced by sex-related neurobiological and developmental factors. Findings revealed distinct brain structure-pain associations in male (*N* = 3,299) and female (*N* = 2,844) youth.
- In males, left sensorimotor cortex thickness was negatively linked to the number of reported pain sites. Pubertal development also moderated several effects, including the cingulate cortical thickness-pain association.
- In females, there was a non-linear relation between surface area of the right supramarginal gyrus and number of pain sites, as it was positively associated only when pain was reported in 3 or more body regions.

**Supplementary Information:**

The online version contains supplementary material available at 10.1186/s13293-026-00898-6.

## Background

Pain is highly prevalent during adolescence [1], yet its underlying mechanisms remain poorly understood, despite significant social and economic impact. Multisite, or widespread, pain is particularly burdensome and difficult to treat, with many adolescents continuing to experience pain into adulthood [2–4]. This persistence may be due to dysregulated pain processing in the central nervous system, known as nociplastic pain [5, 6]. Sex differences in nociplastic pain conditions emerge during adolescence, a critical developmental period marked by puberty [7], when sex-linked shifts in gonadal maturation and secretion of androgens, estradiol, and progesterone shape brain structure and function [8, 9]. Females typically begin puberty before males, although pubertal progression varies widely, even within the sexes [10]. Pubertal development coincides with psychosocial and behavioral changes, and with brain reorganization in pain-related regions, likely increasing the risk of pain onset [11, 12] and contributing to emerging sex differences in nociplastic pain [8].

Although some evidence associates early neurobiological markers with multisite pain in adolescents, research is scarce. Studies in youth show structural changes in areas involved in pain processing that overlap with findings in adults. For example, youth with chronic pain have reduced gray matter volume in the anterior, middle, and posterior cingulate cortex (PCC), reduced cortical thickness in dorsal prefrontal and posterior parietal cortex and increased cortical thickness in PCC, compared to healthy controls [13]. By comparison, meta-analyses in adults with fibromyalgia showed reduced gray matter volume in the left medial prefrontal/anterior cingulate cortex (ACC) and bilateral dorsal PCC, and increased volume in the right pre- and postcentral gyrus and bilateral inferior parietal lobe (IPL) compared to controls [14]. Another meta-analysis across adults with chronic pain conditions found reduced gray matter volume in bilateral insula, right inferior frontal gyrus, ACC, and left superior medial gyrus, and reduced cortical thickness within the left precentral gyrus [15]. Collectively, these findings highlight the potential significance of brain areas including the sensorimotor, the fronto-insular, the cingulate cortex, and the IPL in pain processing across the lifespan, though it is unclear how differences in brain structure relate to multisite pain, differ by sex, or relate to puberty.

Importantly, most studies included in meta-analyses focus on gray matter volume, a composite measure comprising both cortical thickness and surface area. Cortical thickness and surface area, however, are genetically distinct, reflect different neural mechanisms, exhibit limited covariation, and vary by sex [16]. For example, sex-based differences in volume seem to be primarily driven by variations in surface area, rather than cortical thickness, depending on development [17, 18]. Given the sex-related and spatiotemporally unique cortical development of the areas found to be altered in nociplastic pain [19], our study aims to identify cortical thickness and surface area differences related to adolescent multisite pain, separately for males and females.

We hypothesized that cingulate cortex thickness would be inversely related to the number of pain sites across all youth, and that the pattern of relations between brain structure and multisite pain would differ between males and females. We also explored the role of pubertal status as moderator of these associations.

## Methods

### Participants

Participants were selected from the ongoing Adolescent Brain and Cognitive Development (ABCD) Study^®^. Participants provide informed consent (parents) and assent (children), and the study conforms to the rules and procedures of each of 21 sites’ Institutional Review Boards. Protocols for recruitment [20], assessment [21], and imaging [22] have been detailed in previous publications. The ABCD data used in this report include neuroimaging and child and parent reported questionnaires from the Year 2 follow-up visit, when the children were 11–12 years old (release 4.0, NDA Study #1299, DOI: 10.15154/1523041).

**Fig. 1:**
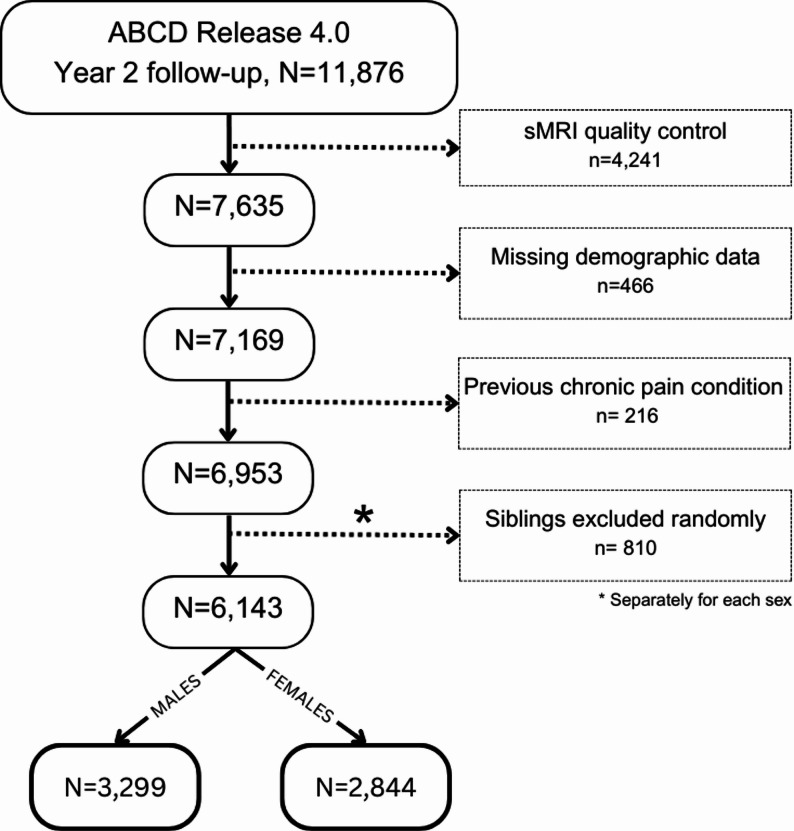
CONSORT diagram showing exclusion criteria

Figure 1 shows the exclusionary steps in a CONSORT diagram. After MRI quality checks, excluding cases with missing relevant demographics and past health conditions which may be related to pain (including cancer/leukemia, cerebral palsy, epilepsy/seizures, lead poisoning, muscular dystrophy, multiple sclerosis, or sickle cell anemia), and selecting one sibling per family, the final sample was *N* = 6,143 (2,844 females).

## Measures

*Pain assessment*: Youth were asked if they had experienced any aches or pains in the past month. If they responded “yes,” they were shown a body map depicting 75 sites and asked to select the specific locations where they experienced pain [23]. This body map is a modified version of the Collaborative Health Outcomes Information Registry (CHOIR), with the mouth as an additional pain site, which was excluded to maintain consistency with the CHOIR body map. We then mapped these 74 sites onto the 2016 American College of Rheumatology body map, a process previously validated in children [24]. Although the range was 0–19 sites, due to the low prevalence of individuals with a higher number of pain sites, scores ≥ 7 were combined into a single score (7+) as the upper limit of this variable (scores > 7 were 1.85% of the sample for both males and females).

*Pubertal status: *The average score of five items from the parent-reported Pubertal Development Scale was used [25]. Questions regarding growth spurt, body hair, and skin changes were asked about both male and female youth. Questions regarding facial hair and voice deepening were asked about male youth, and questions about breast development and menarche were asked about female youth. Each item was rated based on the progression of the characteristic: 1 (has not yet begun), 2 (has barely started), 3 (is definitely underway), and 4 (seems complete). Menarche was either 1 (absent) or 4 (present). A score was calculated only if at least four items were answered, and higher scores indicate more advanced pubertal development.

*Socioeconomic status*: A measure of socioeconomic status (SES), that is, income-to-needs ratio, was calculated by dividing the household income by the corresponding federal poverty line for the reported familial size [26]. A ratio of one represents a household at the federal poverty line, greater than one indicates a family above the federal poverty line, and below one indicates the household is below the federal poverty line.

*Handedness*: Child handedness was coded as either left-handed, right-handed or mixed using a short form of the Edinburgh Handedness Inventory [27] (see Supplementary Measures).

*Age*: Participant's age in months at the 2-year follow-up session was calculated from participant’s birthdate (reported by parents) and the survey date. Age was rounded to the chronological month with 15 days or less coded as 0 months, and 16 days or more coded as 1 month. 

## MRI processing

Tabulated brain structural measures collected from each of the 21 sites (on scanners from 3 different manufacturers) were released by the ABCD Study, after implementing FreeSurfer (version 5.3.0) according to a standardized processing pipeline [22]. The pipeline included skull stripping, white matter segmentation, correction of topological defects, surface optimization, and non-linear registration to a spherical surface-based atlas. Quality control (QC) was accomplished by manually reviewing T1-weighted images and FreeSurfer cortical surface reconstructions. According to ABCD guidelines (MRI Quality Control & Recommended Image Inclusion Criteria), we followed three steps to exclude participants for our investigation: First, we excluded those who did not pass raw T1w image QC criteria according to automated or manual review due to severe artifacts or irregularities (e.g., brain cut-off, wrap-around field of view artifacts). Second, we excluded those flagged with severe artifacts or irregularities during the manual revision of the preprocessed FreeSurfer outputs. Five types of artifacts/problems were considered in this step: motion, intensity inhomogeneity, white matter underestimation, pial overestimation and magnetic susceptibility artifact. Third, we excluded those who did not have tabulated region of interest structural results (e.g., smri_t1w_scs_cbwmatterlh). Volume in mm^3^ of intracranial volume (ICV) as calculated by FreeSurfer (version 5.3.0) was included as a regressor in our analyses [28].

**Fig. 2:**
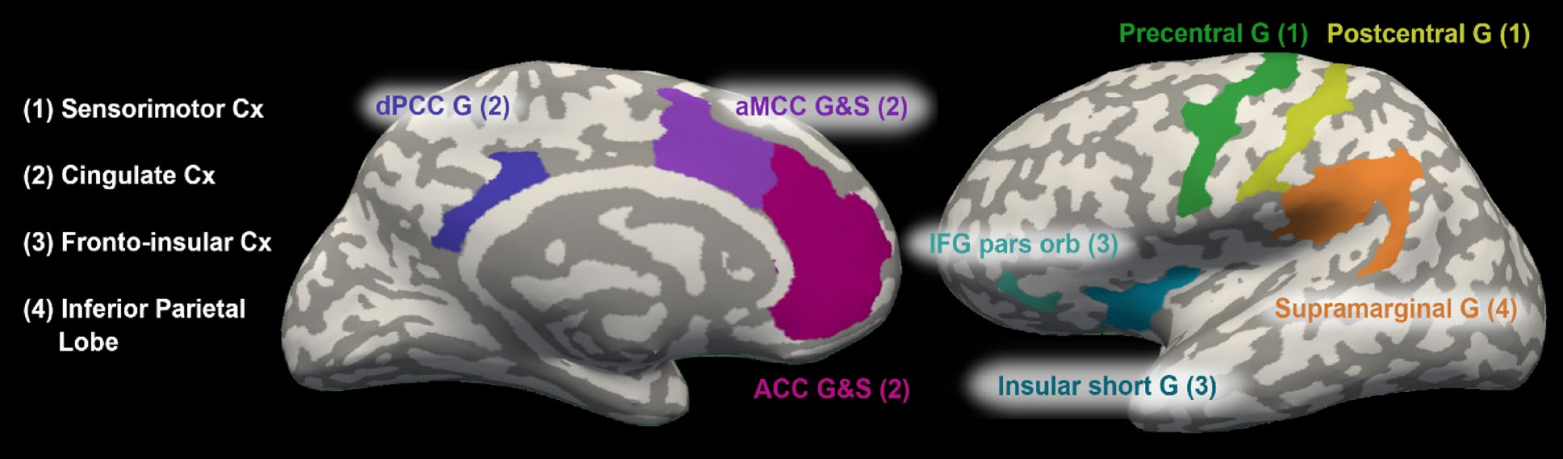
Inflated view of medial and lateral brain surface of one hemisphere displaying each ROI corresponding to: 1. sensorimotor cortex (in green); 2. cingulate cortex (in purple); 3. fronto-insular cortex (in blue); and 4. inferior parietal lobe (in orange).

For analyses, we selected a priori 8 bilateral regions of interest (ROIs), defined by the Destrieux atlas [32] and grouped them according to their anatomical classification and functional characteristics into 4 clusters (see Fig. 2). We leveraged the pediatric and adult literature in our ROI selections, given the small sample sizes (all *N* < 35) in pediatric studies and the partial overlap with findings from adult studies. This selection was based on several factors: morphological characterization of nociplastic pain [29], chronic pain [15], and fibromyalgia [14] in adults and overlapping regions exhibiting links to chronic pain [13, 30] and fibromyalgia [31] in youth (see Table 1). Given the ontogenetic differences between cortical thickness and surface area [18], we examined them separately.

**Table 1:** Definitions of a priori regions of interest used in morphological analyses according to their Destrieux atlas nomenclature, with citations of empirical justifications

Clusters	ROIs	References		Destrieux nomenclature	Description of ROI
		L	*R*		
**Sensorimotor cortex**	Postcentral G	^29^	^14,29^	G_postcentral	Postcentral gyrus
	Precentral G	^15,29^	^14,29^	G_precentral	Precentral gyrus
**Cingulate cortex**	ACC G&S	^15^	^14,15,30^	G_and_S_cingul-Ant	Anterior part of the cingulate gyrus and sulcus (ACC)
	aMCC G&S	^15,30^	^31^	G_and_S_cingul-Mid-Ant	Middle-anterior part of the cingulate gyrus and sulcus (aMCC)
	dPCC G	^14^	^14^	G_cingul-Post-Dorsal	Posterior-dorsal part of the cingulate gyrus (dPCC)
**Fronto-insular cortex**	Insular short G	^15^	^15^	G_insular_short	Short insular gyri
	IFG pars orb	^15^	^15^	G_front_inf-Orbital	Orbital part of the inferior frontal gyrus
**Inferior Parietal Lobe**	Supramarginal G	^13,14^	^14^	G_pariet_inf-Supramar	Supramarginal gyrus

ROI: region of interest; L: Left; R: Right

### Statistical analysis

Analyses were performed in IBM SPSS version 29 [33] and R 4.2.0 [34]. To investigate qualitative or latent sex differences in the neural correlates of adolescent puberty-linked multisite pain, consistent with empirical findings on adolescent pain and theoretical conceptualizations of the study of sex as a biological variable [35, 36], the present analyses were conducted separately for males and females. Assessing the sample as a whole or examining interactive effects with sex instead of using sex-stratified models may occlude these types of differences or contribute to inaccurate reporting of effects (or the lack thereof) due to puberty and sex confounding [35, 36].

To investigate the relation between number of pain sites with cortical structure in each ROI, while accounting for relevant covariates (pubertal status, SES, intracranial volume, race/ethnicity, handedness and age), we employed multilevel modelling, with MRI manufacturer as a random effect. We used Maximum Likelihood (ML) estimation for fitting each model. Specifically, given evidence for non-linear trajectories of adolescent neural development [8, 18], we compared models including linear and quadratic relations of pain sites on brain cortical thickness and surface area (and all covariates). Model selection was performed using Akaike Information Criterion (AIC), in which lower values reflect better relative fit.

Number of pain sites, pubertal status, SES, ICV, and age were used as continuous covariates and were mean-centered, while race/ethnicity and handedness were used as categorical covariates with 5 levels (White [reference], Black, Hispanic, Asian, and other) and 3 levels (right-handed [reference], left-handed, and ambidextrous), respectively. False discovery rate (FDR) correction was applied among the ROIs included in each of the clusters of interest. Although we report all results at *p*<0.05 prior to FDR-correction, only pain associations from the AIC-selected cortical structure models that withstood FDR-correction were interpreted as statistically significant. Finally, exploratory analyses were conducted to assess whether pubertal status moderated the association between linear number of pain sites and cortical structure. Because they were exploratory, p-values were not corrected. For illustrative purposes, the strongest significant interaction was probed applying the Johnson-Neyman technique and using the sim_slopes function in the package *interactions* for R version 4.0.1. [37], which supports multilevel models [38]. This approach calculates the standardized simple slopes and 95% confidence intervals to identify points in the range of values for pubertal status (i.e., the moderator) in which the effect of pain sites is significantly associated with brain structure.

## Results

Demographics of the final sample (*N* = 6,143) are reported in Table 2. Both males and females were mostly right-handed (~ 80%) and around 12 years old on average. Of the total sample, 1.99% were Asian, 11.96% were Black, 19.73% were Hispanic, 55.82% were White, and 10.50% had different race or ethnicity reports (see Supplementary Material). Males (54% of the sample) had less advanced pubertal status compared to females (1.85 vs. 2.36).

**Table 2:** Sample demographics by sex

Sex	Sample size	Number of pain sites: M (SD)	Pubertal status: M (SD)	Age in months: M (SD)	Handedness (%)			Race/ethnicity (%)				
					right	left	ambi	White	Black	Hispanic	Asian	Other
**Males**	*N* = 3,299	1.02 (1.70)	1.85 (0.53)	143.46 (7.78)	79.57	7.12	13.30	57.32	11.58	19.58	1.82	9.70
**Females**	*N* = 2,844	1.04 (1.73)	2.36 (0.66)	142.8 (7.76)	80.84	5.63	13.54	54.08	12.41	19.90	2.18	11.43

M: Mean; SD: Standard Deviation; ambi: ambidextrous

Results for the selected models (linear or quadratic) by sex are shown in Tables S1-S16, including coefficients and AIC values. For both males and females, 28% of the selected models contained number of pain sites as a quadratic term; most were for cortical thickness and area of left hemisphere regions in males, and for cortical thickness in the bilateral sensorimotor cortex in females. Results for each cluster and sex are considered in turn.

**Fig. 3:**
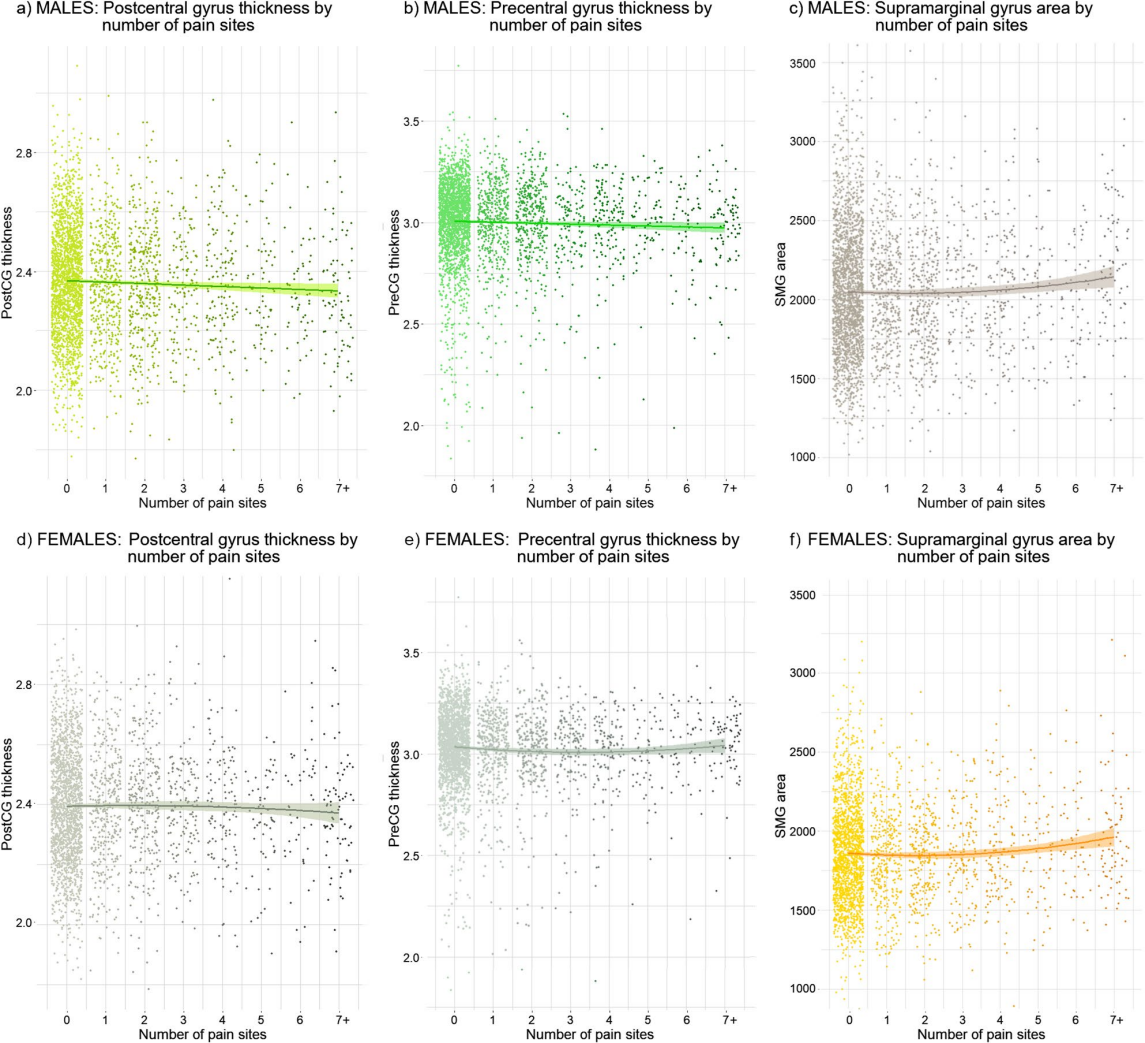
Scatter plots depicting the relation between number of pain sites and cortical measures in males (a,b,c) and females (d,e,f). Each point represents an individual observation (jittered along the x-axis to reduce overlap). Three relations were statistically significant (highlighted in saturated colors). For males, number of pain sites was inversely linearly related to thickness of left postcentral (a) and left precentral (b) gyri. For females, there was a positive quadratic relation between number of pain sites and surface area of the right supramarginal gyrus (f). Plots show unadjusted raw values; statistical inferences are based on FDR-corrected models adjusted for relevant covariates.

Within the sensorimotor cluster, there were significant effects in males that withstood FDR correction, but not in females. Specifically, number of pain sites in males was inversely linearly related to thickness of left post and precentral gyri (post: b=−4.75 × 10^− 3^, SE_b_=1.77 × 10^− 3^, t_(3287)_=−2.68, *p* = 0.007, p_FDR_=0.028; and pre: b=−4.78 × 10^− 3^, SE_b_=2.08 × 10^− 3^, t_(3287)_=−2.41, *p* = 0.016, p_FDR_=0.032, Fig. 3a and b, Table S1). In females, the relation between number of pain sites and cortical thickness of bilateral precentral gyri was best characterized by a quadratic term. Although number of pain sites was positively related to thickness of both left and right precentral gyri (left: b = 2.09 × 10^− 3^, SE_b_=0.97 × 10^− 3^, t_(2831)_ = 2.14, *p* = 0.032, p_FDR_=0.064, Fig. 3e; and right: b = 2.43 × 10^− 3^, SE_b_=1.07 × 10^− 3^, t_(2831)_ = 2.27, *p* = 0.023, p_FDR_=0.064), these associations did not withstand FDR correction (see Table S2).There were no associations for surface area in males or females (S9-S10).

Within the cingulate cortex cluster, surface area associations in males and females were found, but did not withstand FDR correction. In males, there was a negative quadratic relation between number of pain sites and surface area of the left anterior cingulate cortex (ACC: b=−2.02, SE_b_=0.92, t_(3286)_=−2.20, *p* = 0.028, p_FDR_=0.128, Table S11). In females, number of pain sites was positively linearly associated with the surface area of the right ACC (b = 4.29, SE_b_=2.04, t_(2832)_ = 2.10, *p* = 0.036, p_FDR_= 0.159, Table S12). There were no associations for cortical thickness in males or females (Tables S3-S4).

Within the fronto-insular cortex cluster, there were no effects for cortical thickness or surface area in males (Table S5, S13). In females, number of pain sites was inversely linearly associated with the thickness of the inferior frontal gyrus pars orbitalis (IFG: b=−4.35 × 10^− 3^, SE_b_=2.03 × 10^− 3^, t_(2832)_=−2.14, *p* = 0.032, p_FDR_=0.128), but this relation did not withstand FDR correction (Table S6), and there were no associations in surface area (Table S14).

Within the inferior parietal lobe (IPL) cluster, there were no effects in males for cortical thickness or surface area (Tables S7, S15), but there was a significant effect in females that withstood FDR correction. Specifically, there was a positive quadratic relation between number of pain sites and surface area of the right supramarginal gyrus (b = 3.99, SE_b_=1.45, t_(2831)_ = 2.75, *p* = 0.006, p_FDR_= 0.012; Fig. 3f, Table S16). There were no effects in females in cortical thickness (Table S8).

Exploratory analyses revealed interactions between pubertal status and linear number of pain sites for several clusters, primarily in males. The strongest effect was on the thickness of the right anterior mid-cingulate cortex in males (aMCC) (b = 7.73 × 10^− 3^, SE_b_= 2.33 × 10^− 3^, t_(3286)_ = 3.31, *p* < 0.001), but a similar interaction was observed in the left aMCC (b = 4.96 × 10^− 3^, SE = 2.4 × 10^− 3^, t_(3286)_ = 2.01, *p* = 0.045) and the left ACC (b = 4.94 × 10^− 3^, SE_b_=2.18 × 10^− 3^, t_(3286)_ = 2.27, *p* = 0.023). In general, the associations between cingulate cortical thickness and pain sites became increasingly positive with advancing pubertal status. Specifically for the right aMCC, Johnson-Neyman region of significance analyses revealed that male youth with pubertal status scores below 1.73 (i.e., in early puberty) had a negative relation between number of pain sites and cortical thickness, whereas male youth with scores above 2.58 (i.e., beyond mid-puberty) had a positive relation between number of pain sites and cortical thickness (purple regions in Fig. S1a). This interactive effect was not found for females (Fig. S1b). There was, however, a significant interaction for females in the surface area of the left aMCC (b=−4.10, SE_b_=2.03, t_(2831)_=−2.02, *p* = 0.043), with an effect in the opposite direction of males, such that the associations between cingulate cortical thickness and pain sites became increasingly negative with advancing pubertal status.

There were fewer significant interactions in other clusters. Within the sensorimotor cortex, the association between surface area of the left precentral gyrus and pain sites became increasingly inverse with advancing pubertal status in males (b=−9.40, SE = 4.05, t_(3286)_=−2.32, *p* = 0.020). In females, there were no significant interactions in this cluster. Within the fronto-insular cortex cluster, significant interactions were observed in males, specifically for the thickness of the right IFG (pars orbitalis) (b = 7.81 × 10^− 3^, SE = 3.86 × 10^− 3^, t_(3286)_ = 2.02, *p* = 0.043) and the area of the left insular gyrus (b=−2.36, SE = 1.18, t_(3286)_=−2.01, *p* = 0.044). In females, there were no significant interactions in this cluster. Within the IPL cluster, no significant interactions were found for males or females.

## Discussion

**Fig. 4:**
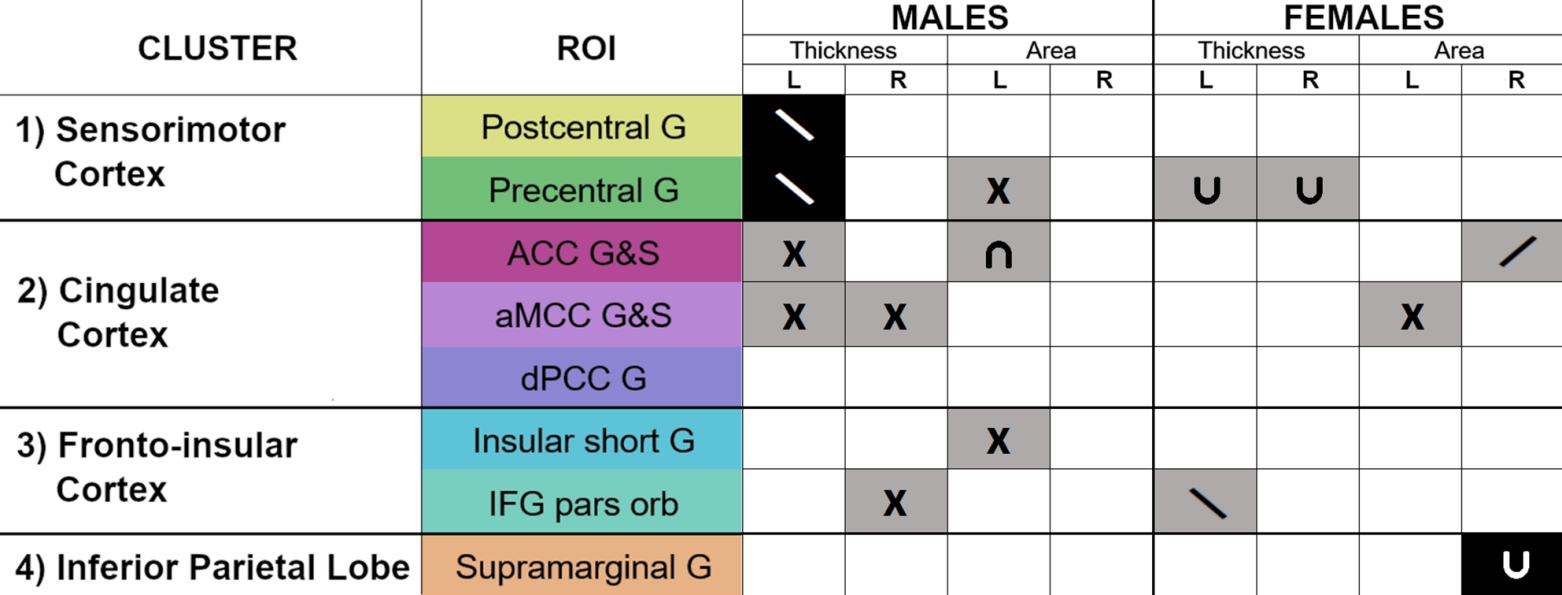
Summary of significant (*p* < 0.05) effects of the number of pain sites on the cortical thickness and area of each of the ROIs, organized by cluster and sex stratified. Uncorrected results are shown in grey, FDR-corrected results are shown in black. (/) denotes a significant positive linear association between number of pain sites and cortical morphology, (\) denotes a significant negative linear association, (U) denotes a significant positive quadratic association, (∩) denotes a significant negative quadratic association, and (x) denotes a significant moderation by pubertal status on the pain-brain structure association

Adolescence is a critical period for neural development and for the emergence of sex differences in pain, but neuroimaging studies on adolescent multisite pain are scant. In this cross-sectional study, we investigated the relation between cortical brain structure and adolescent multisite pain, and we explored the role of pubertal status in moderating this association. Given the sex-related nature of pain and the sex-related nature of pubertal development, we examined relations separately in males and females [36]. Our findings suggest different patterns and/or magnitudes of associations between multisite pain and cortical brain structure in male and female youth, and that puberty moderates brain-pain links especially in males. Although we did not find the hypothesized relation between cingulate cortex thickness and multisite pain, there were differences in this cortical structure for males with multisite pain depending on their pubertal status. A summary of significant (*p* < 0.05) effects of the number of pain sites on the cortical thickness and area of each of the ROIs, including the exploratory analyses, organized by cluster and sex stratified can be found on Fig. 4. This study begins to fill a crucial knowledge gap of the sex-related interplay between nociplastic pain, brain structure, and adolescent development.

Only in males, we observed an inverse relation between cortical thickness of the left sensorimotor cortex and number of pain sites. This is a completely novel finding, as most studies on pain, especially the limited literature on pediatric pain, have samples that are predominantly female. Though novel, this finding is still consistent with the extant literature, given that sensorimotor cortices are core regions for pain processing, and exhibit both structural and functional abnormalities in various chronic pain conditions. For example, children with complex regional pain syndrome have decreased cortical thickness in the precentral gyrus compared to healthy controls [39]. In adults, chronic pain is also associated with cortical thinning in several brain regions [40], including precentral gyri [15, 41]. It is important to note that studies focusing on other structural indices, such as gray matter volume and density, are inconsistent: widespread pain has been associated with an increased volume of sensorimotor cortex, as seen in fibromyalgia [14] and chronic pelvic pain [29], while other chronic pain conditions have decreased gray matter density in these areas compared to healthy controls [42]. Thus, our findings emphasize the importance of cortical thickness (and not just volume generally) in this relation for males, but future work is needed to disentangle the specific associations with different morphometric indices.

In females, we found a non-linear relation between the number of pain sites and the surface area of the right supramarginal gyrus. An increase in volume in this area has been previously reported in adults with fibromyalgia, according to a meta-analysis in which the vast majority of the sample was female [14]. Thus, our study extends this finding to multisite pain in adolescence, potentially marking it as a developmental signature of future chronic pain. Indeed, the inferior parietal lobe, including the supramarginal gyrus, is an important hub in pain processing [43] that exhibits ontogenetic [9] and morphological sex differences. In females, but not in males, surface area decreases over time and females tend to have a greater ratio of gray to white matter than males [44, 45].

Notably, this quadratic pattern observed in females is consistent with a threshold effect: above the approximate inflection point at 3 pain sites, surface area was positively correlated with the number of pain sites. Interestingly, prior work has indicated that 3 or more painful sites spread across multiple body regions (rather than clustered in a single area) [46], is considered multisite or widespread pain, which is suggestive of nociplastic pain mechanisms and is associated with CNS changes, poor sleep and diminished psychological wellbeing [47–49]. While this non-linear association in the supramarginal gyrus was only statistically significant in females, a similar pattern was observed in males and may reflect a quantitative (rather than a qualitative) sex difference [36].

In contrast, although the associations in females within the sensorimotor cortex did not withstand FDR correction, the sex-related differential pattern and direction of the relation between pain and cortical thickness suggest a qualitative sex difference in how multisite pain may influence brain structure in this area [36]. Indeed, there is suggestive evidence for roles of multiple neuroplastic mechanisms–in response to internal and external factors–in this sex difference [50]. Stress stands out as one possibility. The neural impacts of stress follow sex-linked patterns (that also emerge during puberty) and show non-linear associations with stressor duration and gonadal hormone levels. Animal research has revealed that, while chronic stress in male rodents leads to a reduction in neural dendrites across multiple brain regions, in females, it results in an increase in dendritic length and branching—but only in the presence of 17β-estradiol [51]. Future longitudinal studies in youth could examine the unique dynamic relationships between pubertal development, stress and pain in male and female youth.

Cortical thickness and surface area are both influenced by a wide range of genetic and environmental factors, yet they follow distinct developmental trajectories and are shaped by different neurobiological substrates. Whereas surface area is more strongly influenced by early genetic mechanisms and reflects the number of cortical columns, cortical thickness shows greater plasticity across the lifespan and is driven by the number of cells (and their properties) within each cortical column [16] Moreover, the heritability of cortical thickness changes during adolescence (including in the brain regions studied here) [52], and genetic correlations have been observed between surface area and different neuropsychiatric disorders [53].

As shown in prior work, pubertal status can play a crucial role in shaping brain structural changes across development [18, 54, 55], often coinciding with axon pruning and neural apoptosis. The present study is not only consistent with these findings, showing that pubertal status is generally (when significant) negatively associated with cortical thickness and surface area, but also provides preliminary evidence that, especially in males, the relation between pain and cortical structure may be moderated by pubertal development. We observed this moderation most prominently the anterior and middle cingulate cortex, key nodes of the salience network, and that play a crucial role in the cognitive, emotional and motivational dimensions of pain perception [56, 57]. It is worth noting that, despite females having greater variability than males in pubertal status at the ages we investigated, only one marginal puberty effect emerged. This could mean that pubertal status plays a more prominent role in male than female adolescent multisite pain, or, given the sex difference in pubertal onset, it could also mean that the effects occurred in females prior to this assessment, and so, we missed them [58] For male participants still in early puberty, the relation between number of pain sites and thickness of the cingulate cortex was negative, whereas for those beyond mid-puberty, this relation was positive. These findings likely implicate androgens in adolescent pain, consistent with previous work [11, 12].

Both the sex-related patterns of findings in the sensorimotor cortex and these interaction effects may help explain why some studies on chronic pain show decreases in gray matter volume, whereas others show notable increases (including in the somatosensory cortex for chronic pelvic pain patients [29]. Our findings highlight that neuroanatomical changes related to pain can vary by biological factors like sex and pubertal status, helping to reconcile these mixed results. They also raise the possibility that multisite pain may be linked to changes in cortical maturation, potentially accelerating cortical thinning in those still in early puberty and attenuating it in those further along. While speculative, this idea aligns with emerging “brain age” frameworks suggesting accelerated brain aging in chronic pain [59, 60] and highlights the potential influence of puberty and sex on these trajectories. Thus, further longitudinal analyses leveraging pubertal status assessments across multiple timepoints through a neuroendocrinological lens are of utmost importance.

This study has several strengths, including the careful utilization of the large ABCD Study (e.g., considering only one youth per family), a priori ROI selection, and intentional consideration of sex and sex-stratified developmental processes. Social determinants of health were also factored into analyses (i.e., race, ethnicity, and age) and significantly associated with cortical thickness and surface area across multiple brain regions. This study also has some limitations. One is the cross-sectional design (though a necessary starting point for the novel sex-related investigation). Another limitation revolves around the wording of the pain questionnaire, as current and chronic pain are confounded. Future studies that leverage multiple timepoints of ABCD data could address these first two limitations. A third limitation is that at the investigated timepoint of the ABCD Study (11–12 years), males and females are in different stages of pubertal development on average. This is a sex-related feature of development, but it is unclear how much the differences in average pubertal status may have contributed to the sex-stratified patterns we observed. Fourth, though we consider cortical thickness and surface area to be the best indices for our research question, most prior studies have used a voxel-based approach assessing gray matter volume. This hinders direct comparisons of our findings to the extant literature, especially in the context of brain development.

## Conclusions

The present study provides novel insights into how distinct aspects of adolescent brain structure (i.e., cortical thickness and surface area), multisite pain, and pubertal status are interwoven in a sex-dependent manner. We observed unique relations between cortical indices across brain areas and multisite pain for male and female youth, and influences of pubertal status most prominently in males. These findings contribute significantly to understanding how adolescent pain is linked to neural (re)organization, and they lay the groundwork for future longitudinal investigations, and hopefully, opportunities to interrupt the development of chronic pain.

## Supplementary Information

Supplementary Material 1.

## Data Availability

No datasets were generated or analysed during the current study.
