# Supplementary material for: Cortical thickness, surface area, and multisite pain: distinct patterns by sex in adolescence
Source: Biol Sex Differ. 2026 Apr 6;17:108. doi: 10.1186/s13293-026-00898-6 (PMC13181950; doi:10.1186/s13293-026-00898-6)
Supplement: Supplementary file 1 — Supplementary Material 1. [file 13293_2026_898_MOESM1_ESM.docx]

**Supplementary Material**

**Cortical Thickness, Surface Area, and Multisite Pain:**

**Distinct Patterns by Sex in Adolescence**

**Authors:**

Hidalgo-Lopez, E.^1,2^, Portengen, C.^2^, Smith, T.^1^, Becker, H. C.^2^, Schrepf, A.^1^, Harte, S. E.^1^, Beltz, A. M.^2^*, Kaplan, C. M.^1^*

* These authors contributed equally as senior authors.

**Author Affiliations**

^1^Chronic Pain and Fatigue Research Center, Department of Anesthesiology, University of Michigan Medical School, Ann Arbor, MI USA

^2^Department of Psychology, University of Michigan, Ann Arbor, MI USA

Supplement measures:

*Handedness:* Participants were asked to report if they always or usually used their right/left hand, or if they used both hands equally when a) writing, b) throwing, c) using their toothbrush, and d) using a spoon. The response choices included *Always Right*, *Usually Right*, *Both Equally*, *Usually Left*, and *Always Left*. Those answer choices correspond respectively to the numerical values 100, 50, 0, -50, and -100. We used the average composite score as a categorical variable, with 3 categories: right-handed if the mean of these items were higher than 60; left-handed when the mean was lower than -60 ; and ambidextrous for any score in between.

*Race/ethnicity:* At the baseline year, parents reported their child's race/ethnicity by selecting from the following categories: White, Black, Hispanic, Asian, or Other (e.g., Native American or Native Hawaiian/Other Pacific Islander).

Table S1. Sensorimotor cortical thickness in males: Results of mixed effects models. Gray shading indicates the term used in the selected model (linear or quadratic); ^1^ reference: White; ^2^ reference: Right-handed; * indicates p-value<0.05 before FDR-correction.

|  | **mrisdp_28**  **Postcentral G L** | | **mrisdp_102**  **Postcentral G R** | | **mrisdp_29**  **Precentral G L** | | **mrisdp_103**  **Precentral G R** | |
| --- | --- | --- | --- | --- | --- | --- | --- | --- |
| *Predictors* | *Estimates* | *pFDR* | *Estimates* | *pFDR* | *Estimates* | *pFDR* | *Estimates* | *pFDR* |
| (Intercept) | 2.37 | <0.001 | 2.33 | <0.001 | 2.99 | <0.001 | 2.96 | <0.001 |
| Number of pain regions | -0.00 | **0.028*** | -0.00 | 0.282 | -0.00 | **0.032*** | -0.00 | 0.216 |
| Pubertal Status | -0.00 | 0.792 | 0.00 | 0.792 | 0.01 | 0.792 | 0.00 | 0.792 |
| Income to needs | 0.00 | 0.836 | -0.00 | 0.925 | 0.00 | 0.836 | 0.00 | 0.836 |
| ICV | 0.00 | 0.064***** | 0.00 | 0.101 | 0.00 | 0.101 | 0.00 | **0.012*** |
| Black^1^ | -0.09 | **<0.001*** | -0.09 | **<0.001*** | -0.06 | **<0.001*** | -0.06 | **<0.001*** |
| Hispanic^1^ | -0.02 | 0.204 | -0.01 | 0.925 | 0.00 | 0.925 | -0.00 | 0.925 |
| Asian^1^ | -0.06 | **0.020*** | -0.01 | 0.695 | -0.06 | 0.032***** | -0.07 | **0.024*** |
| Other^1^ | -0.04 | **0.002** | -0.05 | **0.002*** | -0.03 | **0.005*** | -0.03 | **0.030*** |
| Left-handed^2^ | -0.00 | 0.771 | -0.01 | 0.771 | -0.00 | 0.771 | 0.01 | 0.771 |
| Ambidextrous^2^ | 0.01 | 0.655 | -0.00 | 0.655 | -0.01 | 0.655 | 0.00 | 0.655 |
| Age | -0.00 | **0.002*** | -0.00 | **0.002*** | 0.00 | 0.065***** | 0.00 | 0.432 |
| Number of pain regions  Quadratic term |  |  |  |  |  |  |  |  |
| Observations | 3299 | | 3299 | | 3299 | | 3299 | |
| AIC | -2252.162 | | -1688.484 | | -1457.811 | | -998.322 | |
| AIC alternative model | -2250.173 | | -1686.505 | | -1456.113 | | -996.475 | |

Table S2. Sensorimotor cortical thickness in females: Results of mixed effects models. Gray shading indicates the term used in the selected model (linear or quadratic); ^1^ reference: White; ^2^ reference: Right-handed; * indicates p-value<0.05 before FDR-correction.

|  | **mrisdp_28**  **Postcentral G L** | | **mrisdp_102**  **Postcentral G R** | | **mrisdp_29**  **Precentral G L** | | **mrisdp_103**  **Precentral G R** | |
| --- | --- | --- | --- | --- | --- | --- | --- | --- |
| *Predictors* | *Estimates* | *pFDR* | *Estimates* | *pFDR* | *Estimates* | *pFDR* | *Estimates* | *pFDR* |
| (Intercept) | 2.41 | <0.001 | 2.37 | <0.001 | 3.03 | <0.001 | 3.00 | <0.001 |
| Number of pain regions | 0.01 | 0.286 | 0.01 | 0.286 | -0.01 | **0.032*** | -0.01 | **0.032*** |
| Pubertal Status | -0.01 | 0.280 | -0.00 | 0.926 | -0.00 | 0.926 | -0.01 | 0.280 |
| Income to needs | 0.00 | 0.106 | 0.00 | 0.106***** | 0.00 | 0.532 | 0.00 | 0.683 |
| ICV | 0.00 | **0.002*** | 0.00 | **0.002*** | 0.00 | **0.041*** | 0.00 | **0.033*** |
| Black^1^ | -0.07 | **<0.001*** | -0.05 | **<0.001*** | -0.06 | **<0.001*** | -0.06 | **<0.001*** |
| Hispanic^1^ | -0.03 | **0.004*** | -0.02 | 0.190 | -0.00 | 0.786 | 0.00 | 0.786 |
| Asian^1^ | -0.04 | 0.348 | -0.02 | 0.556 | -0.03 | 0.556 | -0.00 | 0.910 |
| Other^1^ | -0.04 | **0.004*** | -0.02 | 0.051***** | -0.04 | **0.004*** | -0.02 | 0.090 |
| Left-handed^2^ | -0.01 | 0.787 | -0.01 | 0.797 | 0.00 | 0.883 | 0.01 | 0.797 |
| Ambidextrous^2^ | -0.02 | 0.316 | -0.01 | 0.607 | -0.00 | 0.786 | -0.02 | 0.334 |
| Age | -0.00 | **0.016*** | -0.00 | **0.016*** | 0.00 | 0.454 | 0.00 | 0.363 |
| Number of pain regions  Quadratic term | -0.00 | 0.132 | -0.00 | 0.132 | 0.00 | 0.064***** | 0.00 | 0.064***** |
| Observations | 2844 | | 2844 | | 2844 | | 2844 | |
| AIC | -1876.082 | | -1537.710 | | -1587.688 | | -1050.079 | |
| AIC alternative model | -1875.816 | | -1537.063 | | -1585.114 | | -1046.927 | |

Table S3. Cingulate cortical thickness in males: Results of mixed effects models. Gray shading indicates the term used in the selected model (linear or quadratic); ^1^ reference: White; ^2^ reference: Right-handed; * indicates p-value<0.05 before FDR-correction.

|  | **mrisdp_6**  **ACC G&S L** | | **mrisdp_80**  **ACC G&S R** | | **mrisdp_7**  **aMCC G&S L** | | **mrisdp_81**  **aMCC G&S R** | | **mrisdp_9**  **dPCC G L** | | **mrisdp_83**  **dPCC G R** | |
| --- | --- | --- | --- | --- | --- | --- | --- | --- | --- | --- | --- | --- |
| *Predictors* | *Estimates* | *pFDR* | *Estimates* | *pFDR* | *Estimates* | *pFDR* | *Estimates* | *pFDR* | *Estimates* | *pFDR* | *Estimates* | *pFDR* |
| (Intercept) | 2.88 | <0.001 | 2.86 | <0.001 | 2.87 | <0.001 | 2.92 | <0.001 | 3.11 | <0.001 | 3.11 | <0.001 |
| Number of pain regions | -0.00 | 0.744 | 0.00 | 0.626 | -0.00 | 0.744 | -0.00 | 0.626 | 0.00 | 0.626 | 0.00 | 0.626 |
| Pubertal Status | -0.01 | 0.198 | -0.00 | 0.191 | -0.01 | 0.198***** | -0.00 | 0.483 | 0.01 | 0.191 | 0.01 | 0.191 |
| Income to needs | 0.00 | 0.590 | 0.00 | 0.292 | 0.00 | 0.270 | 0.00 | 0.827 | -0.00 | 0.292 | -0.00 | 0.827 |
| ICV | -0.00 | **0.003*** | -0.00 | **0.003*** | 0.00 | 0.801 | 0.00 | 0.798 | -0.00 | 0.965 | -0.00 | 0.965 |
| Black^1^ | -0.02 | **0.001*** | -0.03 | **0.001*** | -0.05 | **0.001*** | -0.06 | **0.001*** | -0.05 | **0.001*** | -0.03 | **0.002*** |
| Hispanic^1^ | -0.02 | **0.002*** | -0.02 | **0.002*** | -0.02 | **0.014*** | -0.02 | **0.016*** | -0.03 | **0.002*** | -0.02 | **0.039*** |
| Asian^1^ | -0.03 | **0.026*** | -0.01 | 0.802 | -0.01 | 0.784 | 0.00 | 0.813 | 0.02 | 0.802 | 0.01 | 0.802 |
| Other^1^ | -0.02 | **0.005*** | -0.03 | **0.002*** | -0.03 | **0.002*** | -0.02 | **0.003*** | -0.03 | **0.002*** | -0.02 | 0.059 |
| Left-handed^2^ | 0.01 | 0.420 | 0.00 | 0.932 | -0.00 | 0.932 | -0.01 | 0.705 | 0.01 | 0.666 | 0.01 | 0.666 |
| Ambidextrous^2^ | 0.00 | 0.751 | -0.00 | 0.763 | -0.00 | 0.751 | -0.00 | 0.763 | 0.01 | 0.576 | 0.02 | 0.162***** |
| Age | -0.00 | **0.001*** | -0.00 | **<0.001*** | -0.00 | **0.001*** | -0.00 | **<0.001*** | -0.00 | **<0.001*** | -0.00 | **<0.001*** |
| Number of pain regions  Quadratic term | 0.00 | 0.441 |  |  | 0.00 | 0.441 |  |  |  |  |  |  |
| Observations | 3299 | | 3299 | | 3299 | | 3299 | | 3299 | | 3299 | |
| AIC | -4944.556 | | -5035.218 | | -4119.570 | | -4503.049 | | -3299.926 | | -3035.066 | |
| AIC alternative model | -4944.455 | | -5033.219 | | -4119.303 | | -4501.226 | | -3297.929 | | -3033.083 | |

Table S4. Cingulate cortical thickness in females: Results of mixed effects models. Gray shading indicates the term used in the selected model (linear or quadratic); ^1^ reference: White; ^2^ reference: Right-handed; * indicates p-value<0.05 before FDR-correction.

|  | **mrisdp_6**  **ACC G&S L** | | **mrisdp_80**  **ACC G&S R** | | **mrisdp_7**  **aMCC G&S L** | | **mrisdp_81**  **aMCC G&S R** | | **mrisdp_9**  **dPCC G L** | | **mrisdp_83**  **dPCC G R** | |
| --- | --- | --- | --- | --- | --- | --- | --- | --- | --- | --- | --- | --- |
| *Predictors* | *Estimates* | *pFDR* | *Estimates* | *pFDR* | *Estimates* | *pFDR* | *Estimates* | *pFDR* | *Estimates* | *pFDR* | *Estimates* | *pFDR* |
| (Intercept) | 2.89 | <0.001 | 2.86 | <0.001 | 2.88 | <0.001 | 2.93 | <0.001 | 3.10 | <0.001 | 3.12 | <0.001 |
| Number of pain regions | 0.00 | 0.384 | -0.00 | 0.445 | 0.00 | 0.445 | -0.00 | 0.445 | -0.00 | 0.759 | -0.00 | 0.445 |
| Pubertal Status | -0.01 | 0.160 | -0.01 | 0.073 | -0.02 | **0.002*** | -0.01 | **0.002*** | -0.02 | **0.002*** | -0.02 | **0.002*** |
| Income to needs | -0.00 | 0.990 | 0.00 | 0.554 | -0.00 | 0.554 | 0.00 | 0.357 | 0.00 | 0.554 | 0.00 | 0.357 |
| ICV | -0.00 | **0.003*** | -0.00 | **0.003*** | 0.00 | 0.399 | 0.00 | 0.399 | 0.00 | **0.012*** | 0.00 | **0.024** |
| Black^1^ | -0.00 | 0.889 | -0.03 | **0.001*** | -0.04 | **0.001*** | -0.05 | **0.001*** | -0.05 | **0.001*** | -0.05 | **0.001*** |
| Hispanic^1^ | -0.02 | **0.009*** | -0.03 | **0.006*** | -0.01 | 0.078 | -0.01 | 0.106 | -0.01 | 0.078 | -0.02 | **0.018*** |
| Asian^1^ | -0.00 | 0.983 | 0.01 | 0.886 | 0.01 | 0.886 | 0.01 | 0.983 | -0.00 | 0.983 | 0.01 | 0.886 |
| Other^1^ | -0.01 | 0.430 | -0.01 | 0.095 | -0.02 | 0.095 | -0.02 | **0.024*** | -0.01 | 0.317 | -0.02 | **0.027*** |
| Left-handed^2^ | 0.01 | 0.790 | 0.00 | 0.875 | -0.00 | 0.982 | -0.00 | 0.875 | -0.02 | 0.790 | -0.01 | 0.790 |
| Ambidextrous^2^ | -0.01 | 0.170 | -0.01 | 0.170***** | -0.00 | 0.840 | -0.01 | 0.446 | -0.01 | 0.170 | -0.00 | 0.840 |
| Age | -0.00 | **0.001*** | -0.00 | **0.001*** | -0.00 | **0.006*** | -0.00 | **0.001*** | -0.00 | **0.001*** | -0.00 | **0.001*** |
| Number of pain regions  Quadratic term |  |  |  |  |  |  |  |  |  |  |  |  |
| Observations | 2844 | | 2844 | | 2844 | | 2844 | | 2844 | | 2844 | |
| AIC | -4114.442 | | -4344.156 | | -3509.296 | | -3976.319 | | -2931.011 | | -2931.125 | |
| AIC alternative model | -4112.672 | | -4342.160 | | -3507.689 | | -3974.769 | | -2930.222 | | -2929.829 | |

Table S5. Fronto-insular cortical thickness in males: Results of mixed effects models. Gray shading indicates the term used in the selected model (linear or quadratic); ^1^ reference: White; ^2^ reference: Right-handed; * indicates p-value<0.05 before FDR-correction.

|  | **mrisdp_18**  **Insular short G L** | | **mrisdp_92**  **Insular short G R** | | **mrisdp_13**  **IFG pars orb L** | | **mrisdp_87**  **IFG pars orb R** | |
| --- | --- | --- | --- | --- | --- | --- | --- | --- |
| *Predictors* | *Estimates* | *pFDR* | *Estimates* | *pFDR* | *Estimates* | *pFDR* | *Estimates* | *pFDR* |
| (Intercept) | 3.63 | <0.001 | 3.44 | <0.001 | 3.14 | <0.001 | 3.08 | <0.001 |
| Number of pain regions | 0.01 | 0.396 | 0.00 | 0.857 | -0.01 | 0.396 | 0.00 | 0.857 |
| Pubertal Status | -0.01 | 0.348 | -0.02 | **0.016*** | -0.00 | 0.670 | 0.00 | 0.700 |
| Income to needs | 0.00 | 0.236 | 0.00 | 0.216 | 0.00 | 0.244 | 0.00 | 0.911 |
| ICV | -0.00 | **0.002*** | -0.00 | **0.002*** | 0.00 | **0.015*** | -0.00 | 0.553 |
| Black^1^ | -0.02 | 0.306 | 0.01 | 0.391 | -0.02 | 0.306 | -0.01 | 0.391 |
| Hispanic^1^ | -0.02 | 0.083***** | -0.03 | **0.036*** | -0.02 | 0.083 | -0.02 | 0.109 |
| Asian^1^ | -0.04 | 0.236 | -0.03 | 0.247 | -0.03 | 0.236 | -0.05 | 0.216 |
| Other^1^ | -0.00 | 0.768 | 0.01 | 0.785 | -0.03 | **0.020*** | -0.02 | 0.430 |
| Left-handed^2^ | -0.01 | 0.871 | 0.00 | 0.870 | 0.01 | 0.871 | -0.01 | 0.870 |
| Ambidextrous^2^ | -0.00 | 0.955 | -0.00 | 0.970 | -0.02 | 0.224 | -0.00 | 0.970 |
| Age | -0.00 | 0.613 | 0.00 | 0.613 | -0.00 | **0.006*** | -0.00 | **0.004*** |
| Number of pain regions  Quadratic term | -0.00 | 0.168 |  |  | 0.00 | 0.168 |  |  |
| Observations | 3299 | | 3299 | | 3299 | | 3299 | |
| AIC | -1053.421 | | -145.743 | | -1656.112 | | -1182.560 | |
| AIC alternative model | -1052.430 | | -144.387 | | -1654.969 | | -1182.214 | |

Table S6. Fronto-insular cortical thickness in females: Results of mixed effects models. Gray shading indicates the term used in the selected model (linear or quadratic); ^1^ reference: White; ^2^ reference: Right-handed; * indicates p-value<0.05 before FDR-correction.

|  | **mrisdp_18**  **Insular short G L** | | **mrisdp_92**  **Insular short G R** | | **mrisdp_13**  **IFG pars orb L** | | **mrisdp_87**  **IFG pars orb R** | |
| --- | --- | --- | --- | --- | --- | --- | --- | --- |
| *Predictors* | *Estimates* | *pFDR* | *Estimates* | *pFDR* | *Estimates* | *pFDR* | *Estimates* | *pFDR* |
| (Intercept) | 3.62 | <0.001 | 3.43 | <0.001 | 3.16 | <0.001 | 3.11 | <0.001 |
| Number of pain regions | -0.00 | 0.780 | -0.00 | 0.575 | -0.00 | 0.128* | -0.00 | 0.346 |
| Pubertal Status | -0.01 | 0.080***** | -0.03 | **0.004*** | -0.00 | 0.830 | 0.00 | 0.830 |
| Income to needs | 0.00 | 0.080***** | 0.01 | **0.024*** | 0.00 | 0.356 | 0.00 | 0.365 |
| ICV | -0.00 | 0.044***** | -0.00 | **0.004*** | 0.00 | 0.092 | 0.00 | 0.579 |
| Black^1^ | 0.00 | 0.735 | 0.04 | **0.012*** | -0.04 | **0.004*** | -0.02 | 0.212 |
| Hispanic^1^ | 0.01 | 0.660 | -0.03 | **0.033*** | -0.03 | **0.004*** | -0.03 | **0.004*** |
| Asian^1^ | 0.01 | 0.725 | -0.02 | 0.640 | -0.02 | 0.640 | -0.03 | 0.640 |
| Other^1^ | 0.03 | 0.164***** | 0.01 | 0.436 | -0.02 | 0.212 | 0.01 | 0.544 |
| Left-handed^2^ | 0.02 | 0.205 | 0.05 | **0.056** | -0.02 | 0.205 | -0.02 | 0.205 |
| Ambidextrous^2^ | 0.01 | 0.848 | 0.00 | 0.941 | -0.01 | 0.848 | -0.00 | 0.941 |
| Age | -0.00 | 0.057***** | -0.00 | 0.561 | -0.00 | **0.004*** | -0.00 | **0.004*** |
| Number of pain regions  Quadratic term |  |  |  |  |  |  |  |  |
| Observations | 2844 | | 2844 | | 2844 | | 2844 | |
| AIC | -629.527 | | -142.074 | | -1465.703 | | -735.105 | |
| AIC alternative model | -627.727 | | -140.130 | | -1463.772 | | -733.188 | |

Table S7. Inferior parietal lobe cortical thickness in males: Results of mixed effects models. Gray shading indicates the term used in the selected model (linear or quadratic); ^1^ reference: White; ^2^ reference: Right-handed; * indicates p-value<0.05 before FDR-correction.

|  | **mrisdp_26**  **Supramarginal G L** | | **mrisdp_100**  **Supramarginal G R** | |
| --- | --- | --- | --- | --- |
| *Predictors* | *Estimates* | *pFDR* | *Estimates* | *pFDR* |
| (Intercept) | 2.95 | <0.001 | 2.96 | <0.001 |
| Number of pain regions | -0.00 | 0.289 | -0.00 | 0.172 |
| Pubertal Status | 0.00 | 0.952 | 0.00 | 0.952 |
| Income to needs | 0.00 | **0.002*** | 0.00 | 0.269 |
| ICV | -0.00 | 0.800 | 0.00 | 0.278 |
| Black^1^ | -0.04 | **<0.001*** | -0.04 | **<0.001*** |
| Hispanic^1^ | -0.02 | **0.006*** | 0.00 | 0.897 |
| Asian^1^ | -0.01 | 0.996 | 0.00 | 0.996 |
| Other^1^ | -0.04 | **0.002*** | -0.02 | 0.023 |
| Left-handed^2^ | 0.00 | 0.840 | 0.01 | 0.790 |
| Ambidextrous^2^ | -0.00 | 0.883 | 0.00 | 0.883 |
| Age | -0.00 | 0.129 | -0.00 | **0.030*** |
| Number of pain regions  Quadratic term |  |  |  |  |
| Observations | 3299 | | 3299 | |
| AIC | -2954.677 | | -2853.043 | |
| AIC alternative model | -2953.223 | | -2851.150 | |

Table S8. Inferior parietal lobe cortical thickness in females: Results of mixed effects models. Gray shading indicates the term used in the selected model (linear or quadratic); ^1^ reference: White; ^2^ reference: Right-handed; * indicates p-value<0.05 before FDR-correction.

|  | **mrisdp_26**  **Supramarginal G L** | | **mrisdp_100**  **Supramarginal G R** | |
| --- | --- | --- | --- | --- |
| *Predictors* | *Estimates* | *pFDR* | *Estimates* | *pFDR* |
| (Intercept) | 2.97 | <0.001 | 2.98 | <0.001 |
| Number of pain regions | -0.01 | 0.256 | -0.00 | 0.642 |
| Pubertal Status | -0.01 | 0.201 | -0.01 | 0.177 |
| Income to needs | 0.00 | 0.078 | 0.00 | 0.078 |
| ICV | 0.00 | 0.120 | 0.00 | **0.016*** |
| Black^1^ | -0.03 | **0.005*** | -0.04 | **0.002*** |
| Hispanic^1^ | -0.03 | **0.002*** | -0.01 | 0.283 |
| Asian^1^ | 0.01 | 0.688 | -0.05 | 0.034***** |
| Other^1^ | -0.01 | 0.200 | -0.02 | 0.200 |
| Left-handed^2^ | 0.01 | 0.678 | -0.00 | 0.799 |
| Ambidextrous^2^ | 0.01 | 0.672 | -0.01 | 0.524 |
| Age | -0.00 | 0.113 | -0.00 | 0.062***** |
| Number of pain regions  Quadratic term | 0.00 | 0.292 |  |  |
| Observations | 2844 | | 2844 | |
| AIC | -2561.655 | | -2411.859 | |
| AIC alternative model | -2561.539 | | -2409.872 | |

Table S9. Sensorimotor cortical area in males: Results of mixed effects models. Gray shading indicates the term used in the selected model (linear or quadratic); ^1^ reference: White; ^2^ reference: Right-handed; * indicates p-value<0.05 before FDR-correction.

|  | **mrisdp_330**  **Postcentral G L** | | **mrisdp_404**  **Postcentral G R** | | **mrisdp_331**  **Precentral G L** | | **mrisdp_405**  **Precentral G R** | |
| --- | --- | --- | --- | --- | --- | --- | --- | --- |
| *Predictors* | *Estimates* | *pFDR* | *Estimates* | *pFDR* | *Estimates* | *pFDR* | *Estimates* | *pFDR* |
| (Intercept) | 1794.91 | <0.001 | 1602.24 | <0.001 | 1969.29 | <0.001 | 2016.02 | <0.001 |
| Number of pain regions | -2.97 | 0.358 | -1.08 | 0.591 | 2.23 | 0.411 | 3.54 | 0.358 |
| Pubertal Status | -31.62 | **0.002*** | -20.83 | **0.004*** | -26.79 | **0.002*** | -21.70 | **0.005*** |
| Income to needs | 1.72 | 0.589 | -1.21 | 0.559 | 1.79 | 0.589 | 0.94 | 0.600 |
| ICV | 0.00 | **<0.001*** | 0.00 | **<0.001*** | 0.00 | **<0.001*** | 0.00 | **<0.001*** |
| Black^1^ | -62.30 | **0.001*** | -43.63 | **0.001*** | -58.76 | **0.001*** | -41.74 | **0.002*** |
| Hispanic^1^ | -4.35 | 0.676 | -18.26 | 0.733 | -23.10 | 0.050***** | -49.16 | **0.004*** |
| Asian^1^ | -19.20 | 0.941 | -20.73 | 0.941 | -3.86 | 0.941 | -2.16 | 0.941 |
| Other^1^ | 0.14 | 0.991 | -1.91 | 0.991 | -5.22 | 0.991 | -17.46 | 0.784 |
| Left-handed^2^ | -21.49 | 0.506 | -15.17 | 0.506 | -4.08 | 0.848 | -2.88 | 0.848 |
| Ambidextrous^2^ | -4.27 | 0.699 | -10.17 | 0.416 | 12.93 | 0.416 | -16.45 | 0.416 |
| Age | -1.47 | **0.008*** | -1.91 | **0.004*** | -1.09 | **0.041*** | -0.79 | 0.131 |
| Number of pain regions  Quadratic term |  |  |  |  |  |  |  |  |
| Observations | 3299 | | 3299 | | 3299 | | 3299 | |
| AIC | 44788.044 | | 44184.887 | | 44726.503 | | 45012.095 | |
| AIC alternative model | 44789.063 | | 44186.758 | | 44727.684 | | 45014.089 | |

Table S10. Sensorimotor cortical area in females: Results of mixed effects models. Gray shading indicates the term used in the selected model (linear or quadratic); ^1^ reference: White; ^2^ reference: Right-handed; * indicates p-value<0.05 before FDR-correction.

|  | **mrisdp_330**  **Postcentral G L** | | **mrisdp_404**  **Postcentral G R** | | **mrisdp_331**  **Precentral G L** | | **mrisdp_405**  **Precentral G R** | |
| --- | --- | --- | --- | --- | --- | --- | --- | --- |
| *Predictors* | *Estimates* | *pFDR* | *Estimates* | *pFDR* | *Estimates* | *pFDR* | *Estimates* | *pFDR* |
| (Intercept) | 1617.96 | <0.001 | 1445.63 | <0.001 | 1770.03 | <0.001 | 1811.76 | **<0.001** |
| Number of pain regions | -6.10 | 0.528 | 2.42 | 0.536 | -0.48 | 0.818 | 2.19 | 0. 536 |
| Pubertal Status | -27.37 | **0.001*** | -19.14 | **0.001*** | -27.02 | **0.001*** | -12.91 | **0.046*** |
| Income to needs | -0.91 | 0.820 | -1.73 | 0.833 | -0.35 | 0.833 | -0.68 | 0.833 |
| ICV | 0.00 | **<0.001*** | 0.00 | **<0.001** | 0.00 | **<0.001** | 0.00 | **<0.001** |
| Black^1^ | -47.08 | **0.001*** | -39.90 | **0.001*** | -40.24 | **0.001*** | -57.63 | **0.001*** |
| Hispanic^1^ | -9.63 | 0.448 | -6.00 | 0.498 | -17.60 | 0.166 | -29.43 | **0.028*** |
| Asian^1^ | 52.19 | 0.136***** | 35.28 | 0.212 | 21.24 | 0.527 | -14.74 | 0.583 |
| Other^1^ | 9.21 | 0.843 | 0.31 | 0.976 | 8.30 | 0.843 | -6.14 | 0.843 |
| Left-handed^2^ | 5.55 | 0.904 | 17.74 | 0.776 | 1.69 | 0.914 | -6.72 | 0.914 |
| Ambidextrous^2^ | -1.20 | 0.909 | -3.49 | 0.919 | -19.00 | 0.288 | -7.87 | 0.919 |
| Age | -1.53 | **0.004*** | -1.56 | **0.004*** | -0.72 | 0.153 | -0.96 | 0.101 |
| Number of pain regions  Quadratic term | 1.46 | 0.427 |  |  |  |  |  |  |
| Observations | 2844 | | 2844 | | 2844 | | 2844 | |
| AIC | 37905.428 | | 37215.964 | | 37983.097 | | 38401.690 | |
| AIC alternative model | 37905.513 | | 37217.787 | | 37984.106 | | 38402.243 | |

Table S11. Cingulate cortical area in males: Results of mixed effects models. Gray shading indicates the term used in the selected model (linear or quadratic); ^1^ reference: White; ^2^ reference: Right-handed; * indicates p-value<0.05 before FDR-correction.

|  | **mrisdp_308**  **ACC G&S L** | | **mrisdp_382**  **ACC G&S R** | | **mrisdp_309**  **aMCC G&S L** | | **mrisdp_383**  **aMCC G&S R** | | **mrisdp_311**  **dPCC G L** | | **mrisdp_385**  **dPCC G R** | |
| --- | --- | --- | --- | --- | --- | --- | --- | --- | --- | --- | --- | --- |
| *Predictors* | *Estimates* | *pFDR* | *Estimates* | *pFDR* | *Estimates* | *pFDR* | *Estimates* | *pFDR* | *Estimates* | *pFDR* | *Estimates* | *pFDR* |
| (Intercept) | 1802.16 | <0.001 | 2370.39 | <0.001 | 1002.79 | <0.001 | 1098.06 | <0.001 | 523.88 | <0.001 | 454.51 | <0.001 |
| Number of pain regions | 10.54 | 0.135***** | -1.04 | 0.898 | 8.08 | 0.135 | -1.12 | 0.898 | 0.28 | 0.898 | 3.56 | 0.214 |
| Pubertal Status | -23.59 | **0.003*** | -25.78 | **0.003*** | -14.37 | **0.006*** | -13.72 | **0.006*** | -3.81 | 0.238 | -2.76 | 0.309 |
| Income to needs | -0.24 | 0.873 | 1.88 | 0.620 | 0.42 | 0.873 | 1.55 | 0.620 | 0.16 | 0.918 | -0.66 | 0.576 |
| ICV | 0.00 | **<0.001*** | 0.00 | **<0.001*** | 0.00 | **<0.001*** | 0.00 | **<0.001*** | 0.00 | **<0.001*** | 0.00 | **<0.001*** |
| Black^1^ | 11.15 | 0.389 | -19.73 | 0.246 | 15.85 | 0.186 | -5.94 | 0.475 | -5.85 | 0.392 | -12.76 | **0.042*** |
| Hispanic^1^ | -15.46 | 0.164 | -30.39 | **0.012*** | -5.65 | 0.427 | 5.17 | 0.429 | 7.46 | 0.146 | 3.66 | 0.427 |
| Asian^1^ | -9.88 | 0.681 | -122.29 | **0.006*** | 7.48 | 0.681 | -28.32 | 0.269 | -15.98 | 0.269 | 13.81 | 0.257 |
| Other^1^ | 12.62 | 0.514 | -0.62 | 0.961 | 13.56 | 0.315 | 4.95 | 0.654 | 3.31 | 0.654 | 8.63 | 0.315 |
| Left-handed^2^ | -6.16 | 0.764 | -3.55 | 0.800 | -15.21 | 0.306 | 5.13 | 0.768 | -12.17 | 0.198***** | -2.46 | 0.764 |
| Ambidextrous^2^ | -19.62 | 0.111***** | -4.37 | 0.681 | -11.55 | 0.204 | -5.67 | 0.496 | -5.24 | 0.339 | -8.36 | 0.111***** |
| Age | -0.44 | 0.305 | -0.53 | 0.306 | -0.44 | 0.266 | -0.57 | 0.144 | -0.57 | **0.012*** | -0.76 | **0.006*** |
| Number of pain regions  Quadratic term | -2.02 | 0.128***** |  |  | -1.32 | 0.128 |  |  |  |  | -0.72 | 0.128 |
| Observations | 3299 | | 3299 | | 3299 | | 3299 | | 3299 | | 3299 | |
| AIC | 43738.140 | | 44539.184 | | 41843.847 | | 41709.126 | | 38602.791 | | 38016.154 | |
| AIC alternative model | 43740.988 | | 44540.424 | | 41845.484 | | 41711.082 | | 38604.155 | | 38017.590 | |

Table S12. Cingulate cortical area in females: Results of mixed effects models. Gray shading indicates the term used in the selected model (linear or quadratic); ^1^ reference: White; ^2^ reference: Right-handed; * indicates p-value<0.05 before FDR-correction.

|  | **mrisdp_308**  **ACC G&S L** | | **mrisdp_382**  **ACC G&S R** | | **mrisdp_309**  **aMCC G&S L** | | **mrisdp_383**  **aMCC G&S R** | | **mrisdp_311**  **dPCC G L** | | **mrisdp_385**  **dPCC G R** | |
| --- | --- | --- | --- | --- | --- | --- | --- | --- | --- | --- | --- | --- |
| *Predictors* | *Estimates* | *pFDR* | *Estimates* | *pFDR* | *Estimates* | *pFDR* | *Estimates* | *pFDR* | *Estimates* | *pFDR* | *Estimates* | *pFDR* |
| (Intercept) | 1638.46 | <0.001 | 2145.00 | <0.001 | 931.85 | <0.001 | 1003.54 | <0.001 | 458.67 | **<0.001** | 407.91 | <0.001 |
| Number of pain regions | 0.17 | 0.925 | 4.29 | 0.159***** | 1.05 | 0.660 | -0.43 | 0.895 | 1.46 | 0.159 | 1.10 | 0.224 |
| Pubertal Status | -25.01 | **0.002*** | -32.37 | **0.002*** | -14.81 | **0.002*** | -13.17 | **0.002*** | -2.31 | 0.288 | -4.67 | **0.023*** |
| Income to needs | -1.53 | 0.382 | -1.60 | 0.382 | -1.75 | 0.230 | -1.72 | 0.230 | -0.24 | 0.682 | -0.86 | 0.230 |
| ICV | 0.00 | **<0.001*** | 0.00 | **<0.001*** | 0.00 | **<0.001*** | 0.00 | **<0.001*** | 0.00 | **<0.001*** | 0.00 | **<0.001*** |
| Black^1^ | -13.75 | 0.314 | -5.17 | 0.669 | 14.30 | 0.168 | 13.65 | 0.168 | -4.00 | 0.445 | -14.36 | **0.006*** |
| Hispanic^1^ | -16.56 | 0.180 | -19.38 | 0.180 | -5.29 | 0.423 | 9.32 | 0.180 | 5.87 | 0.180 | 5.07 | 0.180 |
| Asian^1^ | 4.11 | 0.968 | -46.79 | 0.168 | -19.42 | 0.464 | -0.64 | 0.968 | 8.02 | 0.563 | 21.82 | **0.054*** |
| Other^1^ | -8.85 | 0.638 | -11.40 | 0.638 | -6.25 | 0.638 | 2.32 | 0.912 | 3.44 | 0.638 | -0.15 | 0.969 |
| Left-handed^2^ | 3.95 | 0.837 | 4.86 | 0.837 | -2.09 | 0.837 | 13.97 | 0.531 | -3.04 | 0.837 | 7.03 | 0.531 |
| Ambidextrous^2^ | -1.40 | 0.905 | 8.19 | 0.905 | -3.25 | 0.905 | 4.44 | 0.905 | 0.46 | 0.905 | -2.59 | 0.905 |
| Age | 0.47 | 0.592 | 0.21 | 0.672 | 0.34 | 0.592 | 0.20 | 0.640 | -0.31 | 0.552 | -0.14 | 0.612 |
| Number of pain regions  Quadratic term |  |  |  |  |  |  |  |  |  |  |  |  |
| Observations | 2844 | | 2844 | | 2844 | | 2844 | | 2844 | | 2844 | |
| AIC | 37290.634 | | 37862.007 | | 35539.502 | | 35428.516 | | 32201.430 | | 31724.535 | |
| AIC alternative model | 37291.722 | | 37863.947 | | 35540.884 | | 35428.765 | | 32203.124 | | 31725.836 | |

Table S13. Fronto-insular cortical area in males: Results of mixed effects models. Gray shading indicates the term used in the selected model (linear or quadratic); ^1^ reference: White; ^2^ reference: Right-handed; * indicates p-value<0.05 before FDR-correction.

|  | **mrisdp_320**  **Insular short G L** | | **mrisdp_394**  **Insular short G R** | | **mrisdp_315**  **IFG pars orb L** | | **mrisdp_389**  **IFG pars orb R** | |
| --- | --- | --- | --- | --- | --- | --- | --- | --- |
| *Predictors* | *Estimates* | *pFDR* | *Estimates* | *pFDR* | *Estimates* | *pFDR* | *Estimates* | *pFDR* |
| (Intercept) | 570.00 | <0.001 | 554.84 | <0.001 | 350.64 | <0.001 | 336.39 | <0.001 |
| Number of pain regions | 0.03 | 0.966 | -0.25 | 0.966 | -2.85 | 0.380 | -0.60 | 0.762 |
| Pubertal Status | -7.83 | **0.004*** | -6.32 | 0.052***** | -1.78 | 0.396 | -4.17 | 0.095 |
| Income to needs | -0.49 | 0.656 | -0.03 | 0.966 | -0.27 | 0.759 | 0.58 | 0.656 |
| ICV | 0.00 | **<0.001*** | 0.00 | **<0.001*** | 0.00 | **<0.001*** | 0.00 | **<0.001*** |
| Black^1^ | -4.70 | 0.289 | -3.07 | 0.540 | -25.68 | **0.002*** | -13.75 | **0.002*** |
| Hispanic^1^ | -5.75 | 0.110 | -1.66 | 0.673 | -10.04 | **0.004*** | -4.75 | 0.181 |
| Asian^1^ | 2.51 | 0.756 | 10.82 | 0.616 | -4.60 | 0.743 | -9.45 | 0.616 |
| Other^1^ | -0.99 | 0.792 | -1.52 | 0.792 | -4.89 | 0.712 | -3.53 | 0.756 |
| Left-handed^2^ | -4.33 | 0.870 | -0.90 | 0.870 | -0.87 | 0.870 | -1.39 | 0.870 |
| Ambidextrous^2^ | -0.87 | 0.783 | 1.17 | 0.783 | -1.71 | 0.785 | -3.90 | 0.783 |
| Age | -0.07 | 0.613 | -0.34 | 0.096 | -0.47 | **0.004*** | -0.37 | **0.032*** |
| Number of pain regions  Quadratic term |  |  |  |  | 0.43 | 0.588 |  |  |
| Observations | 3299 | | 3299 | | 3299 | | 3299 | |
| AIC | 36557.877 | | 38353.467 | | 36335.813 | | 36969.343 | |
| AIC alternative model | 36559.813 | | 38355.467 | | 36335.919 | | 36971.204 | |

Table S14. Fronto-insular cortical area in females: Results of mixed effects models. Gray shading indicates the term used in the selected model (linear or quadratic); ^1^ reference: White; ^2^ reference: Right-handed; * indicates p-value<0.05 before FDR-correction.

|  | **mrisdp_320**  **Insular short G L** | | **mrisdp_394**  **Insular short G R** | | **mrisdp_315**  **IFG pars orb L** | | **mrisdp_389**  **IFG pars orb R** | |
| --- | --- | --- | --- | --- | --- | --- | --- | --- |
| *Predictors* | *Estimates* | *pFDR* | *Estimates* | *pFDR* | *Estimates* | *pFDR* | *Estimates* | *pFDR* |
| (Intercept) | 516.56 | <0.001 | 499.24 | <0.001 | 317.62 | <0.001 | 299.91 | <0.001 |
| Number of pain regions | -2.81 | 0.220 | 0.24 | 0.766 | 0.77 | 0.713 | -3.65 | 0.200 |
| Pubertal Status | -4.79 | **0.032*** | -5.17 | 0.054***** | -3.30 | 0.054 | -3.52 | 0.064 |
| Income to needs | 0.35 | 0.955 | -0.24 | 0.947 | 0.19 | 0.947 | -0.02 | 0.973 |
| ICV | 0.00 | **<0.001*** | 0.00 | **<0.001*** | 0.00 | **<0.001*** | 0.00 | **<0.001*** |
| Black^1^ | -4.94 | 0.241 | -4.46 | 0.359 | -15.35 | **0.004*** | -5.22 | 0.241 |
| Hispanic^1^ | -3.93 | 0.390 | 4.21 | 0.389 | -5.16 | 0.300 | 2.25 | 0.483 |
| Asian^1^ | 0.52 | 0.944 | 5.01 | 0.813 | -17.90 | **0.048*** | -12.73 | 0.212 |
| Other^1^ | -2.19 | 0.538 | 5.95 | 0.273 | -5.16 | 0.273 | -4.98 | 0.273 |
| Left-handed^2^ | 0.74 | 0.874 | 1.13 | 0.860 | 7.10 | 0.444 | 2.61 | 0.874 |
| Ambidextrous^2^ | 0.77 | 0.914 | 6.06 | 0.584 | 0.32 | 0.914 | -1.21 | 0.914 |
| Age | 0.11 | 0.835 | 0.16 | 0.807 | 0.07 | 0.807 | -0.03 | 0.835 |
| Number of pain regions  Quadratic term | 0.44 | 0.304 |  |  |  |  | 0.60 | 0.248 |
| Observations | 2844 | | 2844 | | 2844 | | 2844 | |
| AIC | 31108.228 | | 32679.885 | | 30840.410 | | 31425.770 | |
| AIC alternative model | 31108.277 | | 32681.762 | | 30842.410 | | 31427.248 | |

Table S15. Inferior parietal lobe cortical area in males: Results of mixed effects models. Gray shading indicates the term used in the selected model (linear or quadratic); ^1^ reference: White; ^2^ reference: Right-handed; * indicates p-value<0.05 before FDR-correction.

|  | **mrisdp_328**  **Supramarginal G L** | | **mrisdp_402**  **Supramarginal G R** | |
| --- | --- | --- | --- | --- |
| *Predictors* | *Estimates* | *pFDR* | *Estimates* | *pFDR* |
| (Intercept) | 2479.15 | <0.001 | 2084.34 | <0.001 |
| Number of pain regions | 1.38 | 0.734 | -12.81 | 0.344 |
| Pubertal Status | -40.35 | **0.004*** | -40.15 | **0.002*** |
| Income to needs | 2.21 | 0.488 | 4.37 | 0.196 |
| ICV | 0.00 | **<0.001*** | 0.00 | **<0.001*** |
| Black^1^ | -131.60 | **0.002*** | 6.19 | 0.759 |
| Hispanic^1^ | -28.26 | 0.282 | -1.08 | 0.946 |
| Asian^1^ | -78.73 | 0.258 | -43.80 | 0.307 |
| Other^1^ | -39.02 | 0.210 | 10.75 | 0.589 |
| Left-handed^2^ | -7.50 | 0.779 | -9.64 | 0.778 |
| Ambidextrous^2^ | -28.16 | 0.330 | -13.44 | 0.424 |
| Age | -2.73 | **0.006*** | -2.11 | **0.006*** |
| Number of pain regions  Quadratic term |  |  | 3.16 | 0.108 |
| Observations | 3299 | | 3299 | |
| AIC | 48813.841 | | 47568.011 | |
| AIC alternative model | 48815.132 | | 47569.729 | |

Table S16. Inferior parietal lobe cortical area in females: Results of mixed effects models. Gray shading indicates the term used in the selected model (linear or quadratic); ^1^ reference: White; ^2^ reference: Right-handed; * indicates p-value<0.05 before FDR-correction.

|  | **mrisdp_328**  **Supramarginal G L** | | **mrisdp_402**  **Supramarginal G R** | |
| --- | --- | --- | --- | --- |
| *Predictors* | *Estimates* | *pFDR* | *Estimates* | *pFDR* |
| (Intercept) | 2184.82 | <0.001 | 1885.81 | <0.001 |
| Number of pain regions | 0.19 | 0.959 | -16.87 | 0.084***** |
| Pubertal Status | -37.18 | **0.001*** | -28.31 | **0.001*** |
| Income to needs | -2.34 | 0.770 | 0.79 | 0.734 |
| ICV | 0.00 | **<0.001*** | 0.00 | **<0.001** |
| Black^1^ | -97.72 | **0.002*** | -37.99 | **0.030*** |
| Hispanic^1^ | -28.68 | 0.236 | -4.64 | 0.746 |
| Asian^1^ | -6.35 | 0.888 | 50.10 | 0.312 |
| Other^1^ | -48.08 | 0.052***** | -6.96 | 0.680 |
| Left-handed^2^ | -14.67 | 0.841 | 3.69 | 0.867 |
| Ambidextrous^2^ | 4.58 | 0.810 | 11.96 | 0.812 |
| Age | -2.19 | **0.016*** | -1.81 | **0.017*** |
| Number of pain regions  Quadratic term |  |  | 3.99 | **0.012*** |
| Observations | 2844 | | 2844 | |
| AIC | 41348.437 | | 39956.360 | |
| AIC alternative model | 41350.384 | | 39961.953 | |


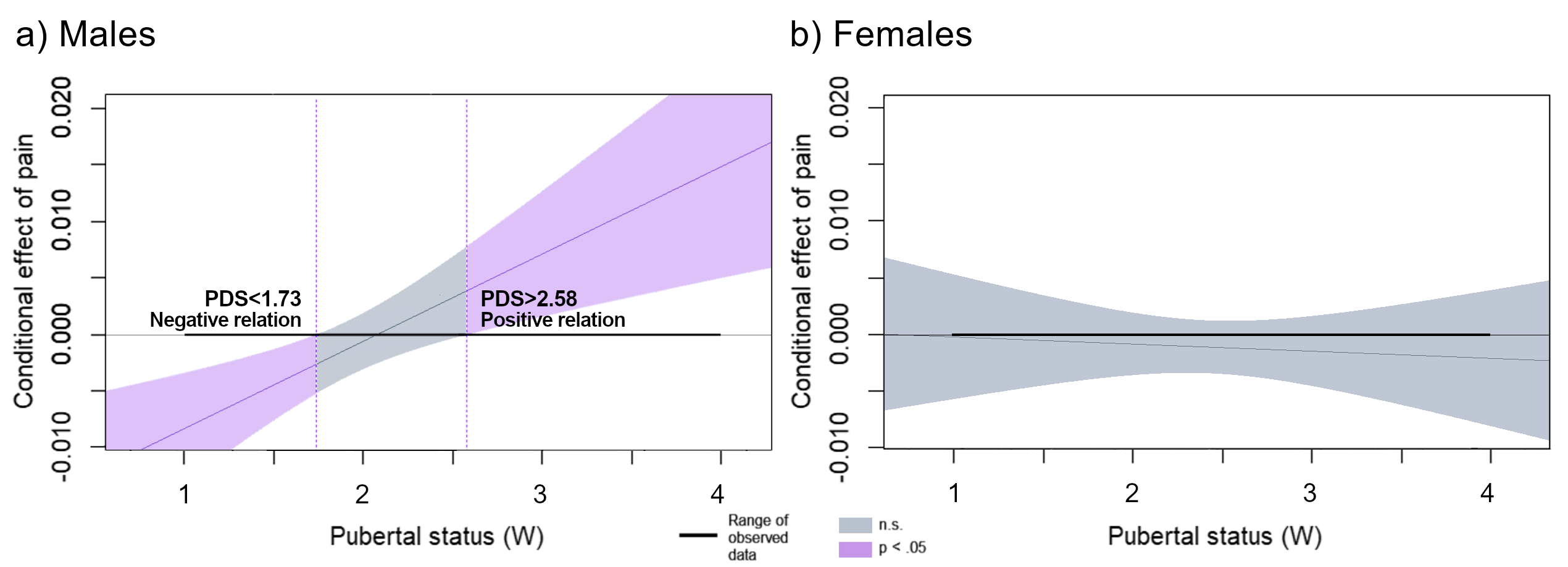


**Figure S1.** Plots illustrating the Johnson–Neyman regions of significance for the relation between number of pain sites and right aMCC cortical thickness at various levels of pubertal status. The X-axis shows pubertal status (assessed by the Pubertal Development Scale, or PDS) values that can range from 1 to 4, and the Y-axis shows the size of the effect (i.e., simple slope) of the relation between number of pain sites and cortical thickness. Only for males (a) was the relation of aMCC cortical thickness with number of pain sites moderated by pubertal status. Below PDS=1.73 and above PDS=2.58, the effect of number of pain sites was significant at *p*<.05 (in purple). This interactive effect was not found for females (b).
